# Delayed Horner’s syndrome and acute stress disorder caused by a large dog bite on the neck: Case report

**DOI:** 10.1097/MD.0000000000040938

**Published:** 2024-12-13

**Authors:** Guoping Dai, Xin Yan

**Affiliations:** a Department of Otorhinolaryngology, Shaoxing People’s Hospital, Shaoxing, China.

**Keywords:** acute stress disorder, case report, delayed Horner’s syndrome, dog bites, psychotherapy

## Abstract

**Rationale:**

The occurrence of delayed Horner’s syndrome caused by a dog bite to the neck is rarely reported. Acute stress disorder (ASD) can easily be neglected when diagnosing this disease in trauma patients who cannot be effectively observed. The symptoms of Horner’s syndrome may not be readily detected in patients with ASD.

**Patient concerns:**

In this report, we present a rare case of a 55-year-old woman with delayed Horner’s syndrome and ASD who initially presented with an internal jugular vein injury caused by a large dog bite on her left neck.

**Diagnosis:**

Delayed Horner’s syndrome and ASD.

**Interventions:**

Neck exploration and internal jugular vein repair were performed under general anesthesia. After the occurrence of Horner’s syndrome and ASD, methylprednisolone and mecobalamin were administered to relieve edema and promote nerve repair, compound anisodine was injected once daily near the left superficial temporal artery to improve microcirculation, escitalopram oxalate and lorazepam were administered to treat anxiety and improve sleep, psychotherapy and narrative nursing were administered once a week.

**Outcomes:**

In the follow-up 4 months at the outpatient clinic after discharge, the patient’s Horner’s syndrome had not healed, but she did not complain of significant discomfort and affected appearance, and ASD did not recur or develop post-traumatic stress disorder.

**Lessons:**

Surgeons should be aware of the possibility of sympathetic nerve injury in patients with deep neck injury, especially in patients with internal jugular vein injury. Peripheral nerves should be explored during the operation. Attention should be paid to the possibility of delayed neurological symptoms and the prevention and treatment of ASD after operation.

## 1. Introduction

Dogs can live and breed as free-living animals, contributing to public health risks.^[1]^ Dog bites are the most common mammalian bites used in emergency departments. Most dog-bite injuries occur in the head and neck regions or extremities.^[2]^ This could lead to physical and psychological harm. Horner’s syndrome is rarely reported among the various types of neck trauma-related complications and was first described in animals by Claude Bernard and in humans by Johann Friedrish Horner in 1869.^[3]^ Although most cases of post-traumatic Horner’s syndrome are diagnosed immediately after a traumatic event, these symptoms may manifest in a delayed manner. In this article, a female patient who suffered from a large dog bite on the left neck and left lower limb is reported. The patient had a tear in the internal jugular vein and developed Horner’s syndrome 1 week after the operation. Acute stress disorder (ASD) was present within 1 month of the bite.

## 2. Case presentation

**Figure 1.**
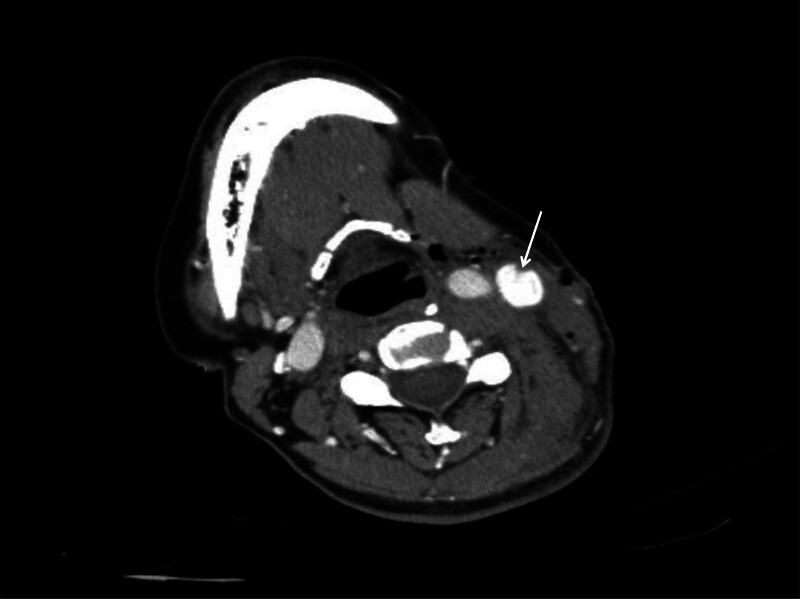
Neck-enhanced CT. The arrow indicates the injury at the left internal jugular vein. CT = computed tomography.

**Figure 2.**
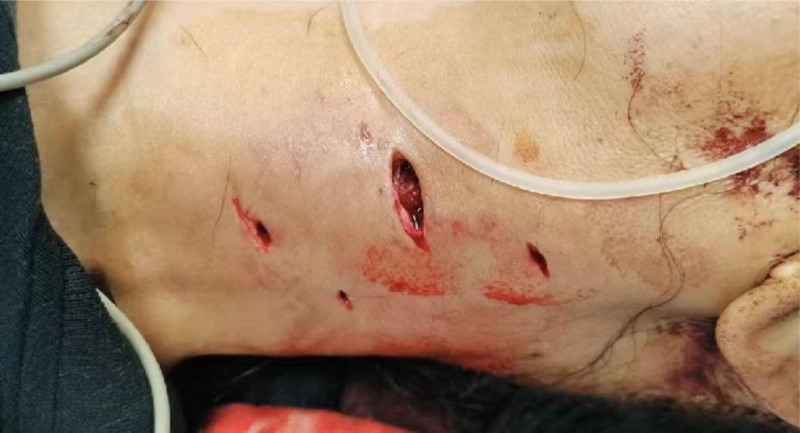
Neck wound, approximately 5, 2, 2, and 1 cm long.

**Figure 3.**
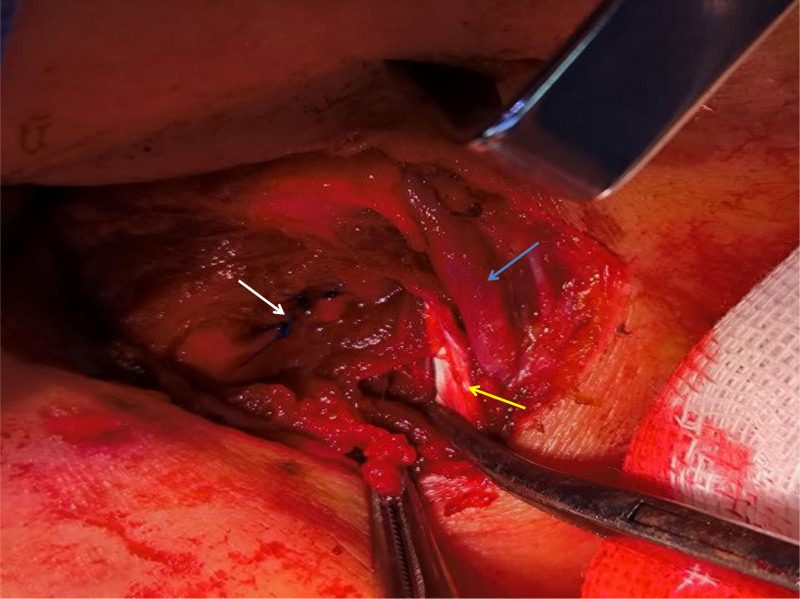
Surgical cavity. White arrow indicates repaired internal jugular vein; yellow arrow indicates damaged sternocleidomastoid muscle; and blue arrow indicates external jugular vein.

**Figure 4.**
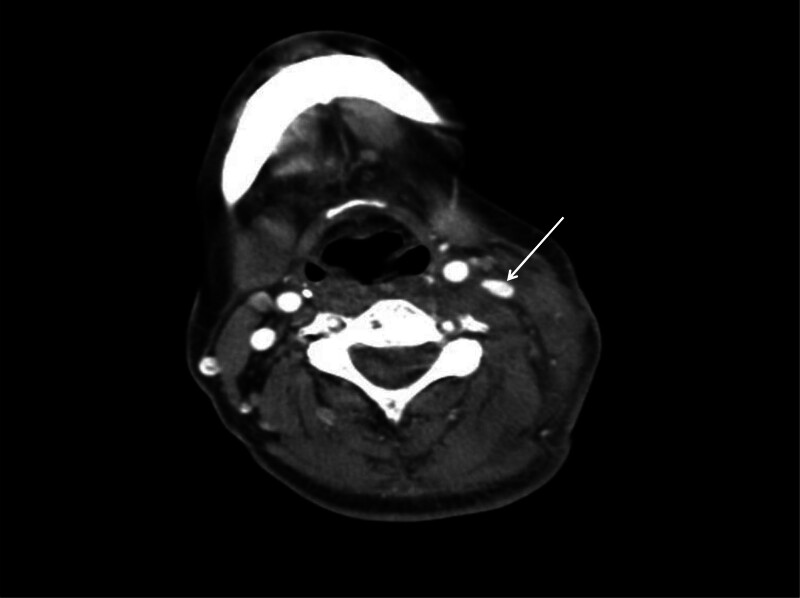
Postoperative enhanced CT at the neck, with the arrow indicating a narrowing of the repaired internal jugular vein diameter and unobstructed blood flow. CT = computed tomography.

**Figure 5.**
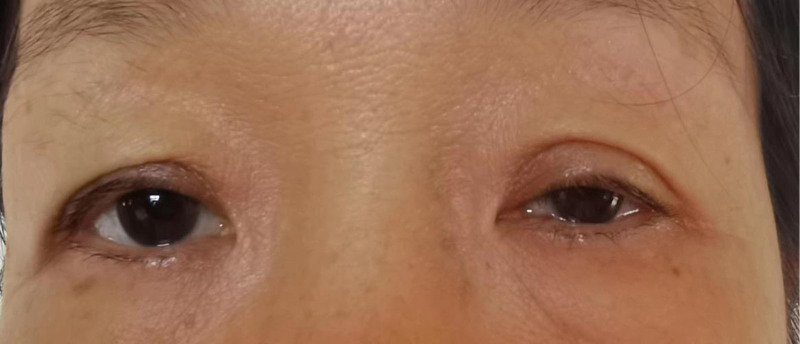
Patient’s left upper drooping eyelid.

**Figure 6.**
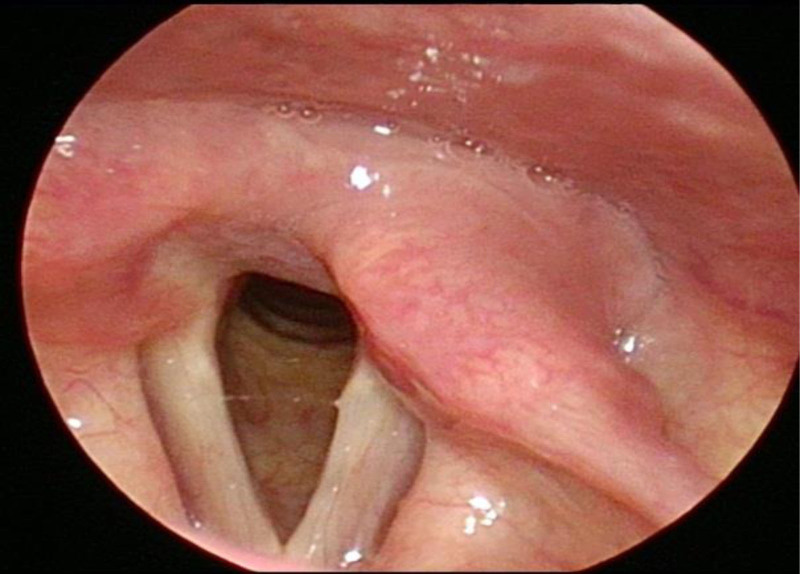
Video laryngoscope indicating good bilateral vocal cord activity.

A 55-year-old woman was sent to the emergency room because of being bitten by a large dog 1 hour prior on the left neck, abdominal wall, and lower limb, followed by syncope for a short time, and extensive bleeding from wounds on the neck and left lower limb. Physical examination revealed a temperature of 36.0°C, blood pressure of 91/59 mm Hg, respiration 19 times/min, heart rate 69 times/min, multiple skin lacerations, and contusions on the left neck, left abdominal wall, and left lower limb. Neck-enhanced computed tomography revealed damage to the left internal jugular vein (Fig. 1). After symptomatic treatment such as fluid replenishment, hemostasis and blood transfusion, probing of the neck, repair of the internal jugular vein, and debridement and suture of the left lower limb were performed under general anesthesia. During the operation, 4 wounds were found on the left neck, approximately 5, 2, 2, and 1 cm long (Fig. 2). After the wound was cleaned, we found a partial tear in the left sternocleidomastoid muscle and a tear of 1 cm in the internal jugular vein, 6-0 prolene suture was performed to close the vascular tear (Fig. 3). Further investigation revealed no other vascular or nerve damages. After the surgery, rabies and tetanus vaccines were administered. Low-molecular-weight heparin was used for anticoagulation, intravenous infusion of cefoperazone sulbactam sodium for anti-infection, methylprednisolone for edema, and mecobalamin tablets for nutritional support. After the injury, the patient repeatedly described a dog bite in tears. She refused to eat meat or even the red food that looked like it. She felt anxious and often experienced dark shadows rushing from the left side, which made it difficult for her to sleep, and often woke up with nightmares of being bitten by dogs at night. After assessment by a psychiatrist, the patient was diagnosed with ASD, and escitalopram oxalate was administered orally to treat anxiety. Oral administration of lorazepam improved anxiety and sleep, and psychotherapy and narrative nursing were administered once a week. One week after the surgery, the patient complained of left upper eyelid ptosis with no sweating on the left face. After ophthalmic consultation, Horner’s syndrome was suspected to have been caused by trauma, and compound anisodine was injected once a day near the left superficial temporal artery to improve microcirculation. Two weeks after the surgery, a neck-enhanced computed tomography scan showed narrowing of the left internal jugular vein and unobstructed blood flow (Fig. 4). The patient was discharged from the hospital after recovering from neck and left lower limb wounds. Her mood and sleep quality improved, but the left upper eyelid ptosis did not. At the outpatient follow-up 4 months after discharge, there was an improvement in sweating on the left side of the face, but no significant improvement in the left upper eyelid ptosis (Fig. 5). Video laryngoscopy revealed good bilateral vocal cord activity (Fig. 6).

## 3. Discussion

Dog bites on the neck often result in open neck injuries. The neck is a densely populated area of important structures, including the respiratory and digestive channels, the thyroid, cervical nerves, and large blood vessels. Severe trauma is often accompanied by damage to important organs or structures and is easily followed by emergency complications such as asphyxia and hemorrhagic shock. If not handled properly, it can lead to sequelae of speech disorders, difficulties in swallowing and breathing, and even endanger lives.^[4,5]^ When dealing with open neck injuries, the first step is to protect the airway and prevent suffocation. Second, it is necessary to actively correct shock, properly manage damaged blood vessels, and maintain stable vital signs. While repairing a wound, it is necessary to explore the surrounding nerves, such as the vagus, recurrent laryngeal, and accessory nerves.

Sympathetic nerve injury can lead to Horner’s syndrome, a clinical syndrome caused by disruption of any part of the ocular sympathetic efferent pathway. It is often characterized by ocular features, such as ptosis and pupillary constriction, with or without sweating on the same side of the face. As the sympathetic nerve is located deep within the prevertebral fascia, it is less likely to be damaged. The most common etiology of Horner’s syndrome is tumor-related (35%–60%), whereas trauma-related Horner’s syndrome is relatively rare (4%–13%).^[6]^ Our patient in this case had Horner’s syndrome 1 week after repair. The reasons are as follows: first, the canine teeth are so sharp that the canine teeth may injure the deep surface of the prevertebral fascia to nerve injury, which is difficult to find during surgical exploration; second, the patient’s condition was masked because of swelling on the left side of the face after the operation, post-injury depression, sleep disorders, and other psychiatric anomalies; therefore, the epiphora was not found in a timely manner, and we speculate that the patient’s sympathetic nerve injury was also caused by the delay of the sympathetic nerve injury due to postoperative tissue edema and its compressive effect. Treatment of Horner’s syndrome mainly focuses on the primary pathology. If the patient has no obvious discomfort, clinical observation is usually performed; if ptosis affects cosmetic appearance, surgical repair is feasible after the stabilization period. Unfortunately, in this case, the patient did not heal 4 months after the trauma, but she did not complain of significant discomfort and affected appearance and received continuous clinical observation.

Patients with trauma are at high risk of developing serious mental health disorders, ASD, and post-traumatic stress disorder. Characterized by dissociative symptoms, subjective re-experiencing, avoidance, and hyperarousal symptoms, ASD is a psychological condition that arises in response to terrifying or traumatic events. Patients develop the disease immediately (within 1 hour) after the onset of stimulation. Symptoms last for a minimum of 3 days, with a maximum of 4 weeks.^[7]^ Therefore, timing plays an important role in the diagnosis of ASD. Veenis al^[8]^ found that 12% of trauma patients discharged within 72 hours or less met the DSM-V diagnostic criteria for ASD. Therefore, it is easy to neglect the diagnosis of this disease in patients with trauma who cannot be effectively observed, and delayed intervention may cause serious psychological harm. After the dog bite, the patient was diagnosed with ASD. Oral drugs relieve anxiety and improve sleep. Psychotherapy and narrative nursing were performed once a week. By listening to and understanding the patient’s story, we can help the patient realize the meaning of the reconstruction of life and disease so that she can actively solve problems and face real life. It includes 4 stages: understanding, feedback, reflection, and witnesses. Gradually, this condition improved through the treatment in 1 month, and this condition did not recur or develop any more post-traumatic stress disorder in the follow-up 4 months at the outpatient clinic after discharge.

## 4. Conclusion

Delayed Horner’s syndrome after trauma is rare. ASD can be also easily neglected in patients with trauma. Therefore, timely intervention and coordination between specialties is key to successful treatment. Understanding this rare clinical course may help surgeons pay attention to not only the early hospital course but also the long-term complications of patients with neck trauma.

## Author contributions

**Conceptualization:** Guoping Dai.

**Data curation:** Guoping Dai, Xin Yan.

**Formal analysis:** Guoping Dai.

**Writing – original draft:** Guoping Dai.

**Writing – review & editing:** Guoping Dai.
